# Effect of High-Tannin and -Polyphenol Plant Material Supplement on Rumen Fermentation, Nitrogen Partitioning and Nutrient Utilization in Beef Cattle

**DOI:** 10.3390/ani14213092

**Published:** 2024-10-26

**Authors:** Pichad Khejornsart, Theerayut Juntanam, Pongsatorn Gunun, Nirawan Gunun, Anusorn Cherdthong

**Affiliations:** 1 Department of Agriculture and Resources, Faculty of Natural Resources and Agro-Industry, Kasetsart University, Chalermphrakiat Sakon Nakhon Provinces Campus, Sakon Nakhon 47000, Thailand; csntyj@ku.ac.th; 2 Department of Animal Science, Faculty of Natural Resources, Rajamangala University of Technology Isan, Sakon Nakhon Campus, Sakon Nakhon 47160, Thailand; pongsatorn.gu@rmuti.ac.th; 3 Department of Animal Science, Faculty of Technology, Udon Thani Rajabhat University, Udon Thani 41000, Thailand; nirawan.gu@udru.ac.th; 4 Tropical Feed Resources Research and Development Center (TROFREC), Faculty of Agriculture, Khon Kaen University, Khon Kaen 40002, Thailand

**Keywords:** tannin-rich tree foliage, ruminal fermentation, nutrients digestibility, nitrogen utilization, tropical beef cattle, microbial protein supply

## Abstract

The current issue of ruminant methane emissions is still being researched by animal nutritionists in an effort to find new approaches. In this study, five beef cattle were randomly assigned in a 5 × 5 Latin square design to examine the effects of supplementation with high-tannin and -polyphenol plant materials on nutrient utilization, rumen fermentation, and nitrogen partitioning. Cattle offered total mixed ration (TMR) silage diets with or without tannin-rich tree leaf or plant herbs, such as *Piper sarmentosum* Roxb., *Cymbopogon citratus* (DC.) Stapf, *Anacardium occidentale* L., and *Careya arborea* Roxb., were supplemented at a dose of 10 g/d. Prior to TMR feeding, the animals’ meals were supplemented with 10 g of fortified plant materials twice a day, along with 100 g of rice bran. The animals in the control group received only 100 g of rice bran and no other plant materials. The result showed that there was no difference in nutrient intake or digestibility between the supplemented and control groups. Although the effect of ruminal pH, NH_3_-N, Total VFA, acetate (C2), and butyrate (C4) was not significant (*p* > 0.05), the proportion of propionate (C3) tended to increase with supplementation (*p* = 0.07). There was no difference in the excretion of purine derivatives or the amount of microbial nitrogen supply, even though supplemented animals had significantly lower protozoal populations than the control group (*p* < 0.05). Moreover, when *A. occidentale* or *C. arborea* was added to the TMR silage diet, the nitrogen intake and retention increased considerably, although total nitrogen excretion decreased. In this approach, the leaves of *Anacardium occidentale* L. and *Careya arborea* Roxb. were particularly promising for strategic supplementation.

## Simple Summary

It may be possible to manipulate rumen fermentation and decrease enteric methane by adding tannin-containing plants to ruminant diet. The effect of tannin-rich tree foliage addition in diets on feed utilization and rumen fermentation characteristics in beef cattle was thus evaluated. This study suggests that feeding 10 g/d of added *Anacardium occidentale* L. or *Careya arborea* Roxb. leaf to beef cattle can reduce protozoal populations and nitrogen excretion without adversely affecting feed utilization or microbial nitrogen supply, regardless of despite the tendency toward improved ruminal fermentation and nitrogen utilization efficiency.

## 1. Introduction

Since climate change is a significant concern as a result of global warming, greenhouse gases may have a significant impact. In addition to that, population growth and dietary changes in humans will continue to be major drivers of the significant increases in the future food demand, and there will likely be an increase in global food consumption over the next decade [1]. This will mostly be caused by the increased use of animal products [2]. Livestock is a critical component of this puzzle because it is the largest land use sector on the planet and can hold many of the solutions to how to feed the world sustainably [3,4]. One of the primary causes of CH_4_ emissions is animal husbandry, which has a warming potential of CO_2_-eq during a 100-year period. Ruminant production is one of the main sources of GHG emissions in agriculture, accounting for 18% to 33% of all methane (CH_4_) emissions into the environment [5,6,7,8]. Ruminant enteric methane, which is thought to be between 80 and 95 million tons, is a significant contributor to global warming [9]. It is because between 2% and 12% of the ruminant’s total energy intake is converted into CH_4_ during rumen fermentation [10]. The effort to decrease anthropogenic GHG emissions must consequently include reducing enteric CH_4_ emissions. Several strategies for reducing enteric methane emissions have been proposed, including feed manipulation, rumen modifiers, and enhancing animal productivity through genetics and management [11,12,13,14,15,16]. Numerous enteric methane (CH4) mitigation opportunities exist to reduce enteric CH4 and other greenhouse gas emissions per unit of product from ruminants. Increased animal productivity resulted in reduced enteric CH4 production per animal production (milk and ADG) and improved feed efficiency [17]. Therefore, it is important to study methane emissions in cattle production since they can lead to the generation of implementable mitigation strategies that can convey economic benefits to farmers, such as a reduction in feed costs, improvement in the animal performance, and productivity in terms of an increase in the production of meat and milk [18].

The comparatively low quality of tropical forages has a significant impact on both the rising intake of fibrous material and the subsequent generation of rumen CH_4_ in ruminant production systems, which are characterized by grazing native and introduced grasses that fluctuate in quantity and quality throughout the year [19]. In the tropical region, there are a variety of resources that might increase animal productivity. A tropical tree consistently finds its means of getting into a ruminant’s diet. One of the main active ingredients in these feed resources is polyphenols or secondary metabolites, such as phenolics, terpenoids, flavonoids, alkaloids, tannins, and saponins [15,20,21,22,23]. Exploiting plant material that contains one or more component polyphenols to alter the rumen microbial community, particularly methanogens, and harness positive rumen fermentation metabolites is thought to be a beneficial approach [24,25]. Governments, researchers, and small-scale farmers, however, may profit from knowledge on the nutritional value, in vivo studies, rumen fermentation efficiency, and fermentation end products of Thai natural tree foliage leaves and herbs because our planet is constantly changing. However, in vivo research based on the supplementation of herbs or tropical tree leaves in ruminants has to be confirmed by many other species, but there is still a restriction in beef cattle. Therefore, the aim of this research was to investigate the tannin-rich tree foliage or plant herbs on the intake, nutrient digestibility, nitrogen utilization, and microbial nitrogen supply of beef cattle.

## 2. Materials and Methods

### 2.1. Animal Management and Design

This research was conducted between September 2021 and January 2022 at the Ani-mal Science Research Unit, Department of Agriculture and Resources, Kasetsart University, Chalermphrakiat Sakon Nakhon Province Campus, Thailand (Northeastern area, Thailand; 17°17′ N, 104°05′ E). All animal experiments were reviewed and approved by Kasetsart University’s Institutional Animal Care and Use Committee (IACUC, No. ACKU64-CSC-004-19/07/2021) in accordance with the National Research Council of Thailand’s Ani-mal Experimentation Ethic.

The experiment included five bull crossbred beef (Thai native × Brahman × Charolais) cattle (12–15 months of age) with an initial body weight (BW) of 218.5 ± 15.78 kg, raised in a 2.5 × 3.0 m individual pen on a cement floor, and there was always access to clean, fresh water and mineral blocks. Animals were treated with ivermectin at a dose of 0.25 mg/kg live body weight at the beginning of the experiment in order to eliminate any parasite. Every animal had a previous vaccination history. All animals were randomly assigned to a 5 × 5 Latin square design with 21 days each period (30 September 2021 to 14 January 2022). Prior to TMR feeding, the animals’ meals were supplemented with 10 g/d of plant material fortification, along with 100 g of rice bran. Only 100 g of rice bran, without any other plant materials, was offered to the animals that represented the control group. TMR silage was offered ad libitum, with two meals each day at 8:00 a.m. and 16:00 *p*.m., with a 10% refusal allowance. During the first 14 days, all animals received their respective treatments, and during the last 7 days, urine and feces were collected for further laboratory analysis. The TMR silage was used as basal diet in a proportion of 40% roughage and 60% concentrate.

The experimental field of Kasetsrt University, Chalermphrakial Sakon Nakhon Province Campus (17°17’ N, 104 05’ E, 172 m above sea level, Sakon Nakhon, Thailand), was used to grow Napier grasses, which were harvested in August 2021 at 45 days of age. Rice straw came from the residues remaining after grain was harvested and obtained from smallholder farmer. Before mixing TMR, Napier grass was manually chopped to a length of 2–3 cm using a forage chopper. The set of equipment included a 70 hp Kubota M7040 tractor (SIAM KUBOTA Corporation, Sakon Nakhon, Thailand) that was used to mix TMR with 500 kg vertical single-auger TMR mixers (108 Agriculture Machine and Equipment Co., Saraburi, Thailand) [20]. A 200 L plastic barrel with a lock-lid that only allowed for gas release was packed with the TMR mixture; 15 barrels were used for each treatment, and the barrels were ensilaged for 21 days before the animal was fed. Table 1 shows the percentage of components and organic acid in TMR silage.

### 2.2. Dietary Treatment Preparation

Nine species were first screened, and only those with the highest nutritional value and the ability to reduce in vitro CH_4_ emissions and the protozoal population when cultured alone were selected [7]. The selected tannin-rich plant and herb samples (leaves and tender stems) were hand collected at local area of Sakon Nakhon Province, Thailand, during the rainy seasons (May–July). The following plant materials were utilized as treatments: *Piper sarmentosum* Roxb., Lemon grass (*Cymbopogon citratus* (DC.) Stapf), Cashew tree leaf (*Anacardium occidentale* L.) and Wild guava leaf (*Careya arborea* Roxb). Fresh and young leaves were taken from Piper sarmentosum Roxb., *Careya arborea* Roxb., *Anacardium occidentale* L., and the 15 cm above the ground of *Cympogon citratus* (DC.) Stapf plants were collected to 5 kg from the farmer pot. All supplementary treatments were then passed through a 0.1 cm screen (Polymix^®^ PX-MFC 90D, Kinematica, Inc., Lucerne, Switzerland) and stored in a plastic zipper at a temperature of −20 °C before being used as dietary treatments.

### 2.3. Data Collection and Chemical Analysis

Animal weights were taken throughout each trial period to regulate the feed intake and maintain a 10% remaining feed level in the trough. Feed and refusal samples were sampled daily during the collection period and were kept at −20 °C for each period prior to analyses. Composites samples were dried at 60 °C and ground (1 mm screen using Polymix^®^ PX-MFC 90D, Kinematica, Inc., Lucerne, Switzerland) and were chemically analyzed for dry matter (DM), ash, ether extract (EE), and crude protein (CP) according to the method of AOAC [26]. Neutral detergent fiber (NDF) and acid detergent fiber (ADF) in substrates were determined according to Van Soest et al. [27] adapted to an Ankom Fiber Analyzer A2000 (Ankom Technology Corp., Fairport, NY, USA). Ether extract content was determined by the Soxhlet method. Tannin-rich tree plant and herbs were analyzed for total polyphenols (TP) by the Folin–Ciocalteu reagent method [28], and condensed tannin (CT) was determined using the HCl-butanol method described by Terrill et al. [29]. Fecal samples were collected twice a day (8:00 and 16:00) during the last three days of each period by using the rectal collection method. Fecal samples were frozen immediately at −20 °C and later dried in a forced-air oven at 60 °C for 48 h and then ground to pass through a 2 mm screen using a Polymix (Polymix^®^ PX-MFC 90D, Kinematica, Inc., Lucerne, Switzerland). Composite fecal matter within cattle and the feed sample were analyzed by using the AIA method [30].

At the end of each period, samples of rumen fluid were taken. Stomach tubing that was attached to a vacuum pump was used to collect approximately 200 mL of rumen fluid, which was then filtered through 4 layers of cheesecloth. The filtrate of ruminal fluid was immediately measured for pH using a portable pH temperature meter (HANNA Instruments HI 9025, Singapore). The initial 50 mL of the mixture was put into a plastic container and mixed with 5 mL of 1 M H_2_SO_4_ before being kept at −20 °C for ammonia nitrogen (NH_3_-N) analyses. Prior to volatile fatty acid (VFA) analysis, a subsample of the mixture was centrifuged at 16,000× *g* for 15 min at 4 °C. The supernatant was then filtered through a 0.45 µm microspore filter and kept at −20 °C. The concentrations of NH_3_-N in rumen fluids were determined using a Kjeltech Auto 1030 (Kjeltech Auto 1030 Analyzer, Tecator, Hoganiis, Sweden) [7], and ruminal VFAs were determined using high-pressure liquid chromatography (Agilent 1200 Series, Agilent Technologies Inc., Santa Clara, CA, USA), with 0.1 M phosphate buffer as mobile phase. The second portion, a 1 mL sample, was immediately added to 9 mL of 10% formaldehyde and stored at 4 °C to be used for total direct counts. A sample was obtained from the supernatant to be counted by a hemacytometer (Boeco, Hamburg, Germany) and a 10× light microscope (Olympus BX51-DIC-B, Olympus Optical Co. Ltd., Hachioji, Tokyo, Japan) in order to determine the protozoa population, which was centrifuged at 1500× *g* for 10 min. In addition to sampling rumen fluid, a blood sample (approximately 10 mL) was also taken from the jugular vein and placed into tubes containing 12 mg of EDTA, then evaluated for blood urea nitrogen (BUN).

Urine samples were collected in plastic cups from tester cattle by spot sampling twice daily for three days after manual stimulation to induce urination (morning and afternoon of the last 3 days of each period). Urine samples were transferred into a 60 mL plastic bottle containing sulfuric acid solution (200 mL/L) and stored at −20 °C. The concentration of creatinine in urine is typically used as an indicator of urinary volume in cattle. Creatinine concentration in urine was analyzed by HPLC, as described by Chen and Gomes [31]. The concentrations of NH_3_-N in urine were determined using a Kjeltech Auto 1030 (Kjeltech Auto 1030 Analyzer, Tecator, Hoganiis, Sweden) [7].

### 2.4. Calculations

The stoichiometric models used for estimating methane [32] and carbon dioxide [33] from VFA composition are as follows:

Methane (CH_4_), mmol/L = 0.45 × acetate − 0.275 × propionate + 0.40 × butyrate (1)

CO_2_ (mol) = (Acetate/2) + (Propionate/4) + (1.5 × Butyrate) (2)

The total amount of internal marker (AIA) throughout the collection period was divided by the concentration of the marker in the composite fecal sample of each animal to estimate total fecal output based on the marker dilution approach. The result was total fecal output in kg per day. The total amounts in kilograms per cattle were calculated by multiplying the total amount of fecal DM excreted per animal by the concentration of each nutrient in the feces.

The method of Chizzotti et al. [34] was used to calculate the total daily urine excretion based on the daily excretion of creatinine and cow body weight. The daily creatinine excretion was determined by multiplying the conversion factor by the body weight of each animal. The concentration of creatinine in the spot urine sample was then multiplied by the daily excretion of creatinine by each animal to obtain the total daily urinary volume excreted.

The excretion of purine derivatives (PDs) was estimated by multiplying the daily urine volume by the concentration of PDs in the urine sample to quantify microbial protein synthesis. The absorbed microbial purines (mmol/day) were calculated from the excretion of PDs (mmol/day), as proposed by Verbic et al. [35], by means of the following equation:

Y = 0.85X + 0.385BW^0.75^ (3)

where: Y, PD excretion (mmol/day); X, PD absorption (once Y is determined, X can be calculated); 0.85, the recovery of absorbed purine as PD in urine (slope); 0.385, beef cattle value; and W^0.75^, the metabolic BW (kg) of the animal.

The intestinal flow of microbial nitrogen compounds (MN, g N/day) was calculated in relation to X, according to Chen and Gomes [31], using the following equation:

MN = 0.727 × [(total PD excretion − 0.385 × BW^0.75^)/0.85] (4)

where: MN, microbial nitrogen; 70, N content in the purines (mg N/mmol); 0.83, digestibility of microbial purines; 0.116, ratio of purine N and total N of rumen microorganisms. The efficiency of microbial N synthesis (EMNS) was calculated by the relationship between the production of MN (g) and the amount of OM digested in the rumen (DOMR). The digestible organic matter in the rumen is calculated by assuming that rumen digestion accounts for 65% of total tract organic matter digestion.

The amount of N consumed (g) was calculated by dividing the amount of CP consumed (g) by 6.25. The total fecal N was determined using the same calculation and the CP values of the feces. The concentration of urea was multiplied by 0.466, which represents the N content in urea, to produce the concentration of urea N in urine, calculating the retained N by subtracting the excreted N (feces + urine) from the N that was consumed. The ratio of N retained devices to N total intake was used to calculate the N utilization efficiency (NUE), as a percentage.

### 2.5. Statistical Analysis

The data were analyzed by using the Statistical Analysis System [36] according to a 5 × 5 Latin square design. The data were analyzed using the statistical model Y_ijk_ = T_i_ + A_j_ + P_k_ + ε_ijk_, where Y_ijk_ is the observation from treatment i, cattle j, and period k; μ is the overall mean; Ti is the mean effect of the treatments (i = 1–5; control, *Piper sarmentosum* Roxb., *Anacardium occidentale* L., *Cymbopogon citratus* (DC.) Stapf, *Careya arborea* Roxb.); A_j_ is the mean effect of the cattle (j = 1–5; the sequent of 5 beef); P_k_ is the mean effect of the periods (k = 1–5; 21 days each period from 30 September 2021 until 15 January 2022); and ε_ijk_ is the residual error. The difference between treatment means was examined using Turkey’s test, and the difference between means was considered statistically significant when the *p*-value was 0.05 or lower.

## 3. Results

### 3.1. Chemical Analyses of TMR Silage and Plant Materials

Table 2 shows the chemical analysis of the plant materials and dietary feed used in this study. The CP contents of the TMR, rice straw, and Napier grass were 12.2%, 2.5%, and 8.5%, respectively, while the NDF contents were 52.4%, 68.7%, and 62.4%, respectively. The total phenol and tannin composition of the TMR contained 0.3% TP and 0.7% CT, respectively. Plant material crude protein levels varied in 1.8% to 15.8% of DM. The highest CP was found in *Piper sarmentosum* Roxb. (15.8% of DM) among all herbs while the lowest in *Cymbopogon citratus* (DC.) Stapf (1.8% of DM). EE was found in small amounts in *Piper sarmentosum* Roxb. (6.4% of DM) followed by *Careya arborea* Roxb. (3.9% of DM). NDF and ADF contents were the lowest in *Anacardium occidentale* L. (45.8 and 39.5% of DM), followed by *Careya arborea* Roxb. (51.4 and 30.9% of DM), and the highest values were found in *Cymbopogon citratus* (DC.) Stapf (67.9 and 42.7% DM). The highest levels of condensed tannin and total phenolic content were found in the leaves of *Anacardium occidentale* L. and *Careya arborea* Roxb., with respective values of 14.2, 15.9, and 19.4, 20.2.

### 3.2. Feed Intake and Nutrient Digestibility

The addition of dietary plant herbs or tannin-rich tree leaves to TMR silage had no effect on feed intake (*p* > 0.05), which varied from 5.99 to 6.18 kg/d, 3.17–3.27% BM, and 117.55–121.14 g/kg BW^0.75^, respectively (Table 3). Consequently, the nutritional intake fractions of DM, OM, CP, EE, NDF, or ADF were unaffected (*p* > 0.05). There was no effect of herbs or tannin-rich tree foliage supplementation on the digestibility of DM, OM, EE, NDF, and ADF. WL tended to decrease the CP digestibility (*p* = 0.08) when compared to the control, reaching 64.62 vs. 62.86%, respectively. The addition of plant material to animals resulted in a considerable increase in CT and TP intake (*p* < 0.05).

### 3.3. Ruminal Fermentation Characteristics, Methane Production and Protozoal Population

The ruminal pH ranged between 6.89 and 6.98, NH_3_-N ranged between 13.50 and 15.51 mg/dl, and TVFA ranged between 97.85 and 110.56 mM; none of these variables changed between treatments (*p* > 0.05, Table 4). There is a tendency for TVFA to decrease when WL supplementation occurs (*p* = 0.08). The proportions of acetate (C2) and butyrate (C4) concentrations were comparable between treatments (*p* > 0.05). A tendency toward a higher propionate (C3) content was seen when WL was supplemented with beef cattle (*p* = 0.07). As a result, the C2:C3 ratio (*p* = 0.08) and CH_4_ (*p* = 0.06) could both decrease linearly post-feeding, while feeding tannin-rich tree leaf and herbs had significantly reduced the protozoal population (*p* < 0.05).

### 3.4. Nitrogen Utilization and Microbial Protein Synthesis

The supplementation of tannin-rich tree leaves and herbs had no influence (*p* > 0.05) on the daily creatinine excretion, blood urea N, urine volume, fecal, and total N consumption (Table 5). Urinary N excretion and fecal N were significantly decreased when beef cattle were fed tannin-rich tree foliage or herb supplementation (*p* < 0.05). Fecal N excretion was similar with CL and WL but lower than that with the control (*p* < 0.05). Thai-native beef cattle fed CL had the lowest urinary and total N excretion (*p* < 0.05), followed by WL and PS. In contrast to N consumption, WL had reduced amounts of fecal and urine N excretion. The proportion of fecal N excretion was the highest in the control group (36.37 g/d), followed by LG (35.59 g/d) and PS (34.78 g/d). Total N excretion was the highest (*p* < 0.05) with the control (78.24 g/d), followed by LG (75.31 g/d) and PS (72.93 g/d). Regarding N retention, the supplementation of tannin-rich tree foliage had a noticeable influence on WL (49.81 g/d) and CL (48.66 g/d) but not on PS (47.66 g/d), and it showed a significant difference from control (*p* < 0.05). The percentage of NUE was the highest (*p* < 0.05) when beef cattle were fed WL (41.51%), followed by CL (40.62%) and PS (39.52%). The amount of allantoin excreted in urine was unaffected when beef cattle were fed tannin-rich tree foliage or herb. NUE, as a percentage of N retained devices to N total intake, was reduced (*p* < 0.05) with CL and WL supplementation, whereas it was not significant to the urinary purine derivative excretion (*p* > 0.05). It was found that the period and animal sequences did not differ significantly in the measured parameters of nitrogen utilization and microbial protein synthesis.

## 4. Discussion

### 4.1. Chemical Analyses of TMR Silage and Plant Materials

The chemical composition of TMR silage was in accordance with a prior composition report [20]. Plant materials are comprised of secondary metabolites, as reported in prior works [7]. The average DM concentration of TMR silage was 34.9%, which may have been a result of the addition of wet cassava pulp and Napier grass. This is comparable with the findings of Kim et al. [37], who reported that 64% of DM that was found in TMR fermented. According to Hao et al. [38], TMR silages preserved for up to 56 days at different moisture levels (40, 45, and 50% as fed) did not change in terms of their chemical composition, including IVDMD, NDF, soluble carbohydrates, CP, and ADF. However, Kondo et al. [39] showed that the storage duration and temperature affected the quantities of soluble protein and NH_3_–N contained in TMR silage.

Enhancing the profitability and productivity of livestock systems in the region and their wide geographical distribution has been made possible by the availability of high-nutrient, easily adoptable, and renewable tropical natural resources, such as grass, legumes, and shrub forage species [14,15,19]. When grazing, the amount of shrubs consumed varies depending on the selectivity and feed preferences of the various animal species; in contrast, cattle are regarded as grazers and prefer grasses. Furthermore, due to the obvious strong interest in CT biological activity in the bovine habitat, it is significant to mention that not all forms of CT are beneficial for ruminant nutrition. Tannins are a diverse group of polyphenolic compounds widely found in various plant species, particularly in tropical regions [40]. Various factors, including species, seasons, and geographical zones, can alter the composition of woody plants, which vary considerably in terms of the nutritional and CT content, as well as methanogenesis capacity [41]. Many trees and bushes keep their leaves throughout the dry season, which reduces the pasture quality while still offering vital nutrients. However, because there is more palatable feed available during the rainy season, when pasture is accessible, cattle may show a lower intake of tree leaves.

### 4.2. Feed Intake and Nutrient Digestibility

Dry matter intake, which is controlled by a variety of parameters, such as chemical compositions and physical and chemical characteristics, can significantly increase animal production [20]. One effective way to enhance feed intake and digestion, particularly in times of fodder scarcity, is to feed cattle tree shrubs and leaves. Many types of trees offer vital nutrients that enhance the health and performance of animals, even in spite of difficulties with tannin content and fiber digestion [21,28,42,43]. The current study’s total DMI was unaffected by the addition of plant materials. However, numerous studies have shown that cattle with high tannin levels voluntarily consume less feed, mostly as a result of decreased nutritional digestibility and palatability [30]. Plants with a high condensed tannin (CT) content (typically >50 g/kg DM) can decrease the voluntary feed intake of ruminants; a moderate or low intake (<50 g/kg DM) has no effect on this [44,45]. According to this study’s results, lowering the CT content was shown to have no effect on the intake or digestibility of beef cattle when plant material was added to the TMR silage diet. On the other hand, by decreasing rumen protein breakdown and raising the post-rumen protein flow, moderate tannin levels, particularly condensed tannins, have demonstrated neutral or even beneficial effects on feed intake [46]. The digestibility of dry matter in tree shrubs is often lower than that of conventional forages like grasses or legumes. However, because tree leaves have a comparatively greater protein content than other feeds, they can improve the quality of the diet when included in a varied diet. Vázquez-Carrillo et al. [47] observed that incorporating *Cymbopogon citratus* (DC.) Stapf into the diets of beef cattle demonstrated positive effects on the feed intake and nutrient digestibility, making it a promising additive in sustainable livestock production. It could be due to the aromatic properties of Lemon grass improving palatability, while its essential oils modulate rumen fermentation and enhance digestibility. Supplementing with Lemon grass, either by itself or in combination with other herbs, decreased protozoa populations and methane output without adversely affecting rumen fermentation or nutrient use [23,47]. According to in vitro research, concentrations of lemongrass essential oil over 25 mg/L may lower the overall number of rumen microorganisms as well as the digestibility of organic matter and dry matter [48]. According to studies by Mandal [49], using *Careya arborea* Roxb. leaves in goat diets at moderate amounts has no detrimental effect on palatability or voluntary feed consumption. *Anacardium occidentale* L. (cashew) has shown potential as a feed supplement for livestock and as a natural alternative to antibiotics in animal nutrition. The leaf contains significant nutrients, with green leaves having the highest crude protein content and in vitro gas production, indicating good digestibility [50]. A mixture including cashew nut shell liquid improved nutrient digestibility and rumen fermentation in beef cattle [51]. In goats, *Piper sarmentosum* Roxb. extract supplementation improved growth performance, antioxidant capacity, and rumen fermentation efficiency, with an optimal dosage of 300 mg/kg showing the best results for average daily gain in goats [52].

### 4.3. Ruminal Fermentation Characteristics, Methane Production and Protozoal Population

The ruminal pH of 6.6 to 6.9, which was within the normal range of 5.5 to 7.0, was a result of the plant material supplementation, while the inclusion of plant materials with a high tannin content such as WL tended to decrease the TVFA concentrations, confirming the findings of earlier studies by Wanapat et al. [23] and Min et al. [24]. It was discovered in earlier research [23] that this considerably reduces protozoal, TVFA, and overall gas production. This result may be connected to the consequence of the quantity of bacteria and TVFA generation. The findings of Cieslak et al. [53] on *Vaccinium vitis* extract supplementation, Wang et al. [45] on *Atractylodes* rhizome and Amur cork tree supplementation, and Chen et al. [54] on tannin are comparable to the trend of the rise in propionate obtained in our study. Methanogenesis and propionate synthesis are two processes that compete for the rumen’s hydrogen metabolism. Therefore, as the results of this study demonstrate, procedures that increase propionate may result in decreased methanogenesis. Additionally, Zhou et al. [52] found that giving goats 1200 mg/kg of *Piper sarmentosum* Roxb. extract decreased the population of fungus, protozoa, *Ruminococcus flavefaciens*, and *Fibrobacter succinogenes* as well as the ratio of acetate to propionate. Carvalho [51] revealed that combining plant extracts and essential oils resulted in a higher concentration of propionate, a lower acetate/propionate ratio, and improved digestibility of dry matter and neutral detergent fiber.

In the rumen, protein breakdown and the deamination of peptides/amino acids produce ammonia-N. Since the addition of high CT and TP of WL somewhat reduced the digestibility of protein, it also decreased the concentration of NH_3_-N in the rumen of beef cattle. Tannins have the advantageous property of preventing rumen protein breakdown, which increases the amount of bypass protein that is accessible for small intestinal digestion [23,28]. This can improve growth and overall protein intake in beef cattle when feed is supplemented with optimal levels of tannin. Despite this, the reduction in rumen NH_3_-N concentrations indicates increased nitrogen utilization by rumen bacteria for MCP production, as well as reduced protein breakdown during rumen fermentation [14,15,20]. Similar to plant herb combinations, Lemon grass, peppermint, and garlic have also demonstrated potential in reducing methane production and protozoal populations in beef cattle without negatively affecting nutrient utilization [23]. Both studies observed decreased methane production and protozoan populations with PS supplementation. Similar effects were noted with sarsaponin, which reduced methane production and protozoal numbers while increasing propionate concentrations in steers [55]. According to Newbold et al. [56], it has been suggested that eliminating all rumen protozoa would increase the supply of microbial protein by 30% and decrease methane production by up to 11%. During ruminal fermentation, enteric CH_4_ is produced and released in higher amounts via eructation than by animal waste. To lower methane production, Dai and Faciola [57] suggested supplementing tannins and using partial rumen protozoa removal strategies. Saponins inhibit protozoa, which reduces the interspecies hydrogen transfer between protozoa and methanogens, thereby lowering the amount of methane produced [14,15,16]. According to reports, cattle given saponins showed a reduction in methane emissions of 10–30% [58]. In this study, the addition of WL (*Careya arborea* Roxb.) seems to minimize the CH_4_ emission impact of protozoal, eliminating up to 65%. A last theory proposes that condensed tannins act as a hydrogen sink themselves, diminishing their availability for carbon dioxide reduction to methane, implying that 1.2 mol methane is reduced per mol of catechin (i.e., 6 H_2_ atoms per molecule of catechin) [59,60].

### 4.4. Nitrogen Utilization and Microbial Protein Synthesis

Generally, dietary proteins can be classified as either degradable or undegradable in the rumen. Rumen bacteria digest a portion of the degradable rumen and utilize it to develop and synthesize microbial proteins [15], which can be lost in the form of urea in urine when the required quantity is exceeded or absorbed by the rumen epithelium in the form of NH_3_. In the current study, the N intake was not affected, and N excretion via urine and feces was reduced by supplementing with both tannin-rich tree leaves and herbs, resulting in an increase in body N retention. As a consequence of increasing retained N, CL and WL supplementation caused a shift in the NUE of beef steer. The results of this study agree with those reported by Aboagye et al. [60], who found that the supplementation of gallic acid and hydrolyzable tannins reduces methane emissions and nitrogen excretion in beef cattle fed a diet containing alfalfa silage. Additionally, nitrogen baling in ruminants is determined by the difference between N intake and N excretion during animal metabolism. Thus, the N provided by the diet, endogenous metabolic N from the oxidation of amino acids, and recycled N for the rumen by blood or saliva are considered N inputs, whereas nutrient outputs include NH_3_, non-degraded protein (dietary or endogenous), and microbial protein excreted via urine and feces [61]. It has the ability to reduce fecal and urinary nitrogen excretion, reducing the environmental impact of beef production, particularly N_2_O, which is released by animal feces and urine [60]. In addition to NH_3_-H reduction, CT and TP may have an effect on *Prevotellaceae*, proteolytic bacteria [45]. The availability of nutrients in plant material stimulates ruminal metabolism, particularly a reduction in protein degradation. This could be attributed, in part, to the availability of plant material, which supplied phytonutrients such as tannins and other polyphenols. Tannins can react with proteins to bind and form complexes that are resistant to ruminal degradation by proteolytic bacteria [15,16,20,44]. This may result in less ammonia being produced and perhaps increase the efficiency of N utilization [58]. This is possible through reductions in protein degradation and by improving the capture efficiency of degraded N in the rumen through microbial protein synthesis, which is related to the availability of energy in the rumen. Despite this, the addition of plant material with high CT and TP had no influence on the microbial protein production of beef cattle.

## 5. Conclusions

The inclusion of a small amount of tannin-rich tree foliage or herbs as supplementation strategies in the present study showed that nutrient digestibility was not influenced compared to the basal diet, and all supplements enhanced nitrogen utilization. The majority of the woody plant supplements evaluated did not have a negative impact on ruminal microbial protein synthesis. Plant materials, such as the leaves of *Anacardium occidentale* L. and *Careya arborea Roxb.*, should be used as primary substrates as strategic supplementation in future investigations. In studies that support tannin-rich tree leaves, the quality of meat and milk must also be assessed in order to support their moderating effects, antioxidant capacity, and environmental intervention in novel scenarios.

## Figures and Tables

**Table 1:** Ingredients of the TMR silage as basal diet with 50:50 roughage to concentrate ratio were used in this experiment.

Items	Ingredients (% DM)
Napier grass (45 day cutting age)	44.5
Rice straw	5.5
Wet cassava pulp	9.0
Cassava chips	15.0
Rice bran	8.4
Palm kernel meal	6.8
Soybean meal	4.0
Mineral mixed *	0.6
Sulfur	0.6
Urea	1.0
Salt	0.6
Molasses	4.0
The Quality of TMR silage, % DM (*n* = 3)	
pH	4.39
Lactic acid	6.13
Acetic acid	0.85
Butyric acid	0.62
Propionic acid	1.24
NH_3_-N	1.89

* Mineral (each kg contain): calcium = 164.00 g, cobalt = 0.04 g, copper = 1.00 g, iodine = 0.04 g, iron = 2.00 g, magnesium = 2.89 g, manganese = 11.00 g, phosphorus = 80.00 g, selenium = 0.03 g, sodium = 136.60 g, sulfur = 19.20 g and carrier = 1000.00 g.

**Table 2:** Chemical composition of TMR silage, rice straw, Napier grass and supplemental treatment (n = 3).

Variation	%DM							
	DM	OM	CP	EE	NDF	ADF	CT	TP
*Piper sarmentosum* Roxb., PS	24.8	92.1	15.8	6.4	64.7	39.8	8.9	9.1
*Cymbopogon citratus* (DC.) Stapf, LG	25.1	89.7	1.8	1.9	67.9	42.7	1.5	2.5
*Anacardium occidentale* L., CL	23.9	92.3	9.6	1.2	45.8	39.5	14.2	19.4
*Careya arborea* Roxb., WL	24.6	91.4	15.0	3.9	51.4	30.9	15.9	20.2
TMR silage	34.9	91.0	12.2	9.2	52.4	31.5	0.3	0.7
Rice straw	92.8	91.5	2.5	0.4	68.7	45.6	-	-
Napier grass	17.2	89.7	8.5	1.9	62.4	32.8	0.5	1.2

OM, organic matter; CP, crude protein; EE, ether extract; NDF, neutral detergent fiber; ADF, acid detergent fiber; CP, crude protein; CT, condensed tannins; TP, total phenol.

**Table 3:** Total feed intake and digestibility of nutrients as affected by supplementation of tannin-rich tree foliage or herbs in beef cattle.

Items	Control	PS	LG	CL	WL	SEM	*p*-Value		
							Trt	Period	Animal
Feed intake									
DM, kg/d	6.18	6.02	6.11	5.98	5.99	0.268	0.67	0.21	0.47
%BW	3.27	3.19	3.21	3.18	3.17	0.127	0.61	0.35	0.45
g/kg BW^0.75^	121.14	118.30	119.34	117.74	117.55	4.684	0.47	0.33	0.38
Nutrients intake, kg DM/d									
OM	5.71	5.56	5.64	5.53	5.53	0.268	0.25	0.18	0.12
CP	0.77	0.75	0.76	0.75	0.75	0.039	0.23	0.25	0.34
EE	0.18	0.17	0.18	0.17	0.17	0.185	0.14	0.15	0.14
NDF	3.35	3.26	3.30	3.24	3.24	0.175	0.27	0.42	0.47
ADF	2.13	2.08	2.11	2.06	2.07	0.092	0.28	0.67	0.48
CT, g/d	0.017 ^c^	0.019 ^ab^	0.018 ^bc^	0.019 ^ab^	0.020 ^a^	0.0008	0.03	0.12	0.09
TP, g/d	0.040 ^b^	0.043 ^ab^	0.043 ^ab^	0.044 ^a^	0.044 ^a^	0.0010	0.02	0.10	0.11
Digestibility coefficients, %									
DM	65.45	64.94	65.03	65.74	64.89	1.465	0.98	0.46	0.98
OM	70.01	69.65	69.91	70.16	69.07	1.390	0.99	0.39	0.97
CP	64.62	62.22	64.62	63.72	62.86	0.893	0.08	0.93	0.89
EE	63.05	64.28	64.86	63.50	63.29	2.154	0.54	0.54	0.47
NDF	58.51	58.12	58.33	57.71	57.91	2.073	0.95	0.73	0.95
ADF	43.12	43.51	42.97	44.34	41.17	2.511	0.93	0.11	0.32

^a,b,c^ means within row showed with different superscript letter accepted significantly different; OM, organic matter; CP, crude protein; EE, ether extract; NDF, neutral detergent fiber; ADF, acid detergent fiber; CP, crude protein; CT, condense tannins; TP, total phenol; PS, *Piper sarmentosum* Roxb.; LG, Lemon grass (*Cymbopogon citratus* (DC.) Stapf); CL, Cashew leaf (*Anacardium occidentale* L.); WL, Wild guava leaf (*Careya arborea* Roxb.); SEM, standard error of the mean; Trt, Treatment.

**Table 4:** Effect of supplementation of tannin-rich tree foliage or herbs in beef cattle fed TMR silage on rumen ecology, fermentation efficiency, and protozoal population.

Items	Control	PS	LG	CL	WL	SEM	*p*-Value		
							Trt	Period	Animal
pH	6.89	6.78	6.98	6.90	6.94	0.060	0.16	0.11	0.21
NH_3_-N (mg/dL)	15.51	14.86	13.53	13.50	12.42	0.131	0.06	0.23	0.14
Total VFA (mM)	107.9	104.02	110.56	99.78	97.85	3.493	0.08	0.15	0.09
VFA (mol/100 mol)									
Acetate (C2)	68.59	67.29	67.89	66.98	65.97	3.023	0.20	0.57	0.84
Propionate (C3)	20.27	21.77	21.13	21.04	22.86	1.087	0.07	0.19	0.23
Butyrate (C4)	11.14	10.95	10.98	11.98	11.17	0.238	0.14	0.28	0.39
C2:C3	3.38	3.09	3.21	3.18	2.89	0.147	0.08	0.31	0.18
CH_4_, mmol/L	29.75	28.67	29.13	29.15	27.17	0.951	0.06	0.10	0.13
CO_2_, mol	56.07	55.50	55.69	56.72	55.46	0.384	0.17	0.26	0.34
Protozoal, 10^5^ cell/mL	5.75 ^a^	2.14 ^b^	1.95 ^b^	2.95 ^b^	2.01 ^b^	0.988	0.02	0.08	0.10

^a,b^ means within row showed with different superscript letter accepted significantly different; OM, organic matter; CP, crude protein; EE, ether extract; NDF, neutral detergent fiber; ADF, acid detergent fiber; CP, crude protein; CT, condensed tannins; TP, total phenol; PS, *Piper sarmentosum* Roxb.; LG, Lemon grass (*Cymbopogon citratus* (DC.) Stapf); CL, Cashew leaf (*Anacardium occidentale* L.); WL, Wild guava leaf (*Careya arborea* Roxb.); SEM, standard error of the mean; Trt, Treatment.

**Table 5:** Effect of supplementation of tannin-rich tree foliage or herbs in beef cattle fed TMR silage on nitrogen utilization and urinary purine derivative.

Items	Control	PS	LG	CL	WL	SEM	*p*-Value		
							Trt	Period	Animal
Creatinine, mg/dL	28.48	27.03	29.94	29.82	28.13	0.981	0.44	0.98	0.42
Blood Urea N, mg/dL	13.18	13.54	13.02	13.05	12.40	0.154	0.37	0.94	0.27
Urinary Volume, L/d	7.09	6.86	7.15	6.95	7.05	0.844	0.52	0.85	0.78
Fecal Output, kg DM/d	2.29	2.28	2.44	2.38	2.26	0.241	0.59	0.39	0.91
Total N Intake, g/d	123.88	120.59	122.34	119.79	119.99	2.192	0.48	0.54	0.46
Urinary N, g/d	41.86 ^a^	38.16 ^b^	39.72 ^ab^	37.06 ^b^	37.44 ^b^	0.874	0.02	0.09	0.14
Fecal N, g/d	36.37 ^a^	34.78 ^bc^	35.59 ^ab^	34.08 ^bc^	32.74 ^c^	0.718	0.03	0.32	0.17
Total N Excretion, g/d	78.24 ^a^	72.93 ^bc^	75.31 ^ab^	71.14 ^c^	70.18 ^c^	1.045	0.05	0.13	0.45
N Retention, g/d	45.64 ^a^	47.66 ^ab^	47.03 ^ab^	48.66 ^bc^	49.81 ^c^	0.947	0.04	0.18	0.15
NUE, %	36.84 ^c^	39.52 ^b^	38.44 ^bc^	40.62 ^ab^	41.51 ^a^	0.624	0.05	0.10	0.29
Urinary purine derivatives									
Allantoin, mmol/d	123.81	119.88	120.45	123.19	120.50	2.840	0.79	0.28	0.33
PD excretion, mmol/d	135.64	128.68	129.3	131.97	129.31	4.509	0.58	0.21	0.86
PD absorption, mmol/d	108.30	105.83	107.68	105.76	105.27	5.195	0.54	0.29	0.27
MN supply, g N/d	78.73	76.94	78.28	76.89	76.53	2.619	0.53	0.58	0.56
EMNS, g N/kg DOMR	21.08	21.28	21.11	21.41	21.27	0.453	0.62	0.61	0.47

SEM, standard error of the mean; PS, *Piper sarmentosum* Roxb.; LG, Lemon grass (*Cymbopogon citratus* (DC.) Stapf); CL, Cashew leaf (*Anacardium occidentale* L.); WL, Wild guava leaf (*Careya arborea* Roxb.); Trt, Treatment; Different letters on the row indicate significance at 5% (*p* < 0.05). NUE, nitrogen utilization efficiency; PD, purine derivative; MN, microbial nitrogen; EMNS, efficiency of microbial nitrogen synthesis; DOMR, digestible of organic matter in the rumen (if rumen digestion 65% of digestion in total tract); DOMR = DOMI × 0.65; DOMI, digestion organic matter intake; Urinary purine derivative contained allantoin 80–85%, Calculated from (PD excretion − 0.147 × BW^0.75^)/0.85.

## Data Availability

Data are available upon request to the corresponding author.
